# Evaluation of tumor motion effects on dose distribution for hypofractionated intensity‐modulated radiotherapy of non‐small‐cell lung cancer

**DOI:** 10.1120/jacmp.v11i3.3182

**Published:** 2010-06-08

**Authors:** Hyejoo Kang, Ellen D. Yorke, Jie Yang, Chen‐Shou Chui, Kenneth E. Rosenzweig, Howard I. Amols

**Affiliations:** ^1^ Department of Medical Physics Memorial Sloan‐Kettering Cancer Center New York NY 10065 USA; ^2^ Sun Yat‐Sen Cancer Center Taipei Taiwan; ^3^ Department of Radiation Oncology Memorial Sloan‐Kettering Cancer Center New York NY 10065 USA

**Keywords:** hypofractionation, lung cancer, IMRT, organ motion effects

## Abstract

Respiration‐induced tumor motion during intensity‐modulated radiotherapy (IMRT) of non‐small‐cell lung cancer (NSCLC) could cause substantial differences between planned and delivered doses. While it has been shown that, for conventionally fractionated IMRT, motion effects average out over the course of many treatments, this might not be true for hypofractionated IMRT (IMHFRT). Numerical simulations were performed for nine NSCLC patients (11 tumors) to evaluate this problem. Dose distributions to the Clinical Target Volume (CTV) and Internal Target Volume (ITV) were retrospectively calculated using the previously‐calculated leaf motion files but with the addition of typical periodic motion (i.e. amplitude 0.36–1.26 cm, 3–8 sec period). A typical IMHFRT prescription of 20 Gy × 3 fractions was assumed. For the largest amplitude (1.26 cm), the average ± standard deviation of the ratio of simulated to planned mean dose, minimum dose, D95 and V95 were 0.98±0.01, 0.88±0.09, 0.94±0.05 and 0.94±0.07 for the CTV, and 0.99±0.01, 0.99±0.03, 0.98±0.02 and 1.00±0.01 for the ITV, respectively. There was minimal dependence on period or initial phase. For typical tumor geometries and respiratory amplitudes, changes in target coverage are minimal but can be significant for larger amplitudes, faster beam delivery, more highly‐modulated fields, and smaller field margins.

PACS number: 87.55.dk

## I. INTRODUCTION

Highly conformal photon dose distributions generated with intensity‐modulated radiation therapy (IMRT) often improve the therapeutic ratio, permitting higher tumor doses while respecting normal tissue tolerance. Recently there has been increasing use of hypofractionated IMRT (IMHFRT) at our institution^(^ ^1^ ^)^ and others^(^ ^2^ ^)^ for treatment of inoperable early stage non‐small‐cell lung cancer (NSCLC) using treatment schedules such as 30 Gy × 1 fraction,^(^ ^3^ ^,^ ^4^ ^)^ 20 Gy × 3 fractions,^(^ ^5^ ^)^ 15 Gy × 3 fractions,^(^ ^6^ ^)^ 12 Gy × 4 fractions.^(^ ^7^ ^)^ Early studies report better local control than conventional multi‐fractionated treatments, with acceptable morbidity.

The amplitude of lung tumor respiratory motion is typically 0.5–2.5 cm with periods of 3–8 seconds.^(^ ^8^ ^,^ ^9^ ^,^ ^10^ ^)^ To improve dose coverage of the gross and clinical tumor volume (GTV and CTV) an internal target volume (ITV) is often defined, but dose calculations usually do not account for motion‐related effects such as interplay and blurring.

Blurring^(^ ^11^ ^,^ ^12^ ^,^ ^13^ ^,^ ^14^ ^,^ ^15^ ^,^ ^16^ ^,^ ^17^ ^)^ refers to changes in dose to a target voxel caused by motion to a region, where the dose is very different from what was planned. It is dependent on respiration amplitude and the degree of modulation in the plan. For target voxels near the beam penumbra, blurring results in dose reduction even for non‐IMRT treatments. IMRT interplay refers to a change in delivered dose caused by tumor motion relative to MLC leaf motion. During delivery, a target voxel, assumed to be stationary for treatment planning dose calculations, may move relative to the moving MLC leaves and receive a significantly different dose. Interplay effects also increase with respiratory amplitude, and may also depend on the breathing period and the breathing phase at the start of each beam. For identical treatment plans, the expectation value of a patient's dose distribution is a function mostly of blurring, while statistical variation is mostly determined by interplay.

Previous investigations^(^ ^12^ ^,^ ^13^ ^,^ ^14^ ^,^ ^15^ ^)^ showed that blurring and interplay effects average out for IMRT consisting of >10 fractions. For IMHFRT, however, there are far fewer treatment fractions but more breathing cycles per treatment field and the statistics are very different. This treatment planning study attempts to answer whether these averaging effects also result in only small perturbations to delivered dose for IMHRFT.

## II. MATERIALS AND METHODS

### A. Treatment Plans

Dose distributions from the clinical treatment plans for nine early‐stage NSCLC patients (11 tumors) previously treated at our institution with IMHFRT (20 Gy × 3 or 12 Gy × 4 fractions) were retrospectively recalculated to assess perturbations in delivered doses resulting from respiratory motion. At simulation, patients were immobilized in a customized body cradle, and free breathing plus respiration correlated (4DCT) planning scans were acquired. The physician delineated the GTV from the planning scan and expanded it to an ITV using the 4DCT. The CTV was defined as ITV plus 0 to 5 mm margin and the PTV encompassed the CTV with 5 mm margin in all directions. Treatment plans were designed to give full dose coverage to the PTV while respecting departmental normal tissue constraints: maximum spinal cord dose (≤ 24Gy/3 fractions), ipsilateral lung ($V_{20}$ (percentage of structure receiving >20% of the prescribed dose) ≤25%), total lungs ($V_{20}$ ≤12%), and the mainstem and distal bronchi (maximum dose ≤30 Gy/3 fractions and 60 Gy/3 fractions, respectively). At each treatment fraction, a kilovoltage cone beam CT (kVCBCT) was acquired and the soft tissue in the GTV region was registered to the planning CT for patient setup at each treatment fraction. Department policy limits IMHFRT to patients whose respiratory motion amplitude, tumor location and size are appropriate for the ensuing larger ITVs. Our department's technique for lung IMHFRT typically consists of 3–7 coplanar 6 MV sliding window IMRT beams, concentrated on the ipsilateral lung, and delivered using a Varian MLC with 5 mm leaf width running at a dose rate of 600 MU/min. For the past year, we have taken care to ‘spread out’ the beams to reduce skin toxicity.^(^ ^1^ ^)^ This differs from the ≥ ten‐field, non‐coplanar, static field technique used by many others but, after more than two years of experience, our technique appears to have similar local control/complication outcomes.^(^ ^18^ ^)^

All calculations were done on an in‐house treatment planning system^(^ ^19^ ^,^ ^20^ ^)^ (written in Fortran and C++, and currently running on a networked system of Windows‐based PCs with ~4 GB of memory and high end video cards). A radiological path‐length corrected pencil beam algorithm is used for tissue inhomogeneity correction. The IMRT optimization algorithm uses an iterative gradient search method to minimize a quadratic objective function that includes target dose uniformity and normal tissue maximum dose, mean dose and dose‐volume constraints.^(^ ^21^ ^)^ A research module modifies the intensity profile incident on a tissue voxel to account for relative motion between the voxel and the MLC as described below.

The NSCLC IMHFRT treatments typically require relatively modest beam modulation, but we also examined the effect of respiratory motion on treatment plans with more highly‐modulated beams.

### B. Respiratory motion simulation

We used the methods of Chui et al.,^(^ ^13^ ^)^ which simulate one dimensional tumor motion, either parallel or perpendicular to MLC leaf motion. Motion in lung is primarily in the cranial‐caudal direction and perpendicular to leaf motion, which is typically in the axial plane.^(^ ^22^ ^,^ ^23^ ^)^ Thus, respiration can displace a tumor voxel from beneath its planned leaf pair to an adjacent pair where it will receive a different dose. Respiratory motion parallel to leaf motion exposes a voxel to the open portion of the leaf pair for a different amount of time than planned, also resulting in different delivered doses. This study concentrates and reports in detail on motion perpendicular to the leaves, although effects of periodic parallel motion were also investigated.

**Figure 1:**
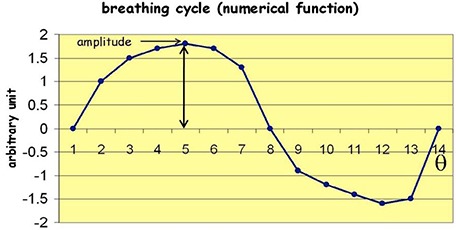
Function used to simulate respiratory motion with an arbitrary unit of amplitude with 13 equal‐spaced phases (Θ) in time.

The total beam intensity received at a point, X (x,y) is the integral over time or monitor units (MU) of the product of the intensity $I(X{-}_{\chi l,k}(t))$ for the *k*th left leaf and the intensity $I(X{-}_{\chi l,k}(t))-X)$ for the *k*th right leaf (x and y coordinates parallel to and perpendicular to leaf motion). In this formula, ${\;}_{\chi l,k}$ and ${\;}_{\chi r,k}$ are the locations of the *k*th left and right MLC leaves where *k* is determined by the point's y coordinate. For stationary voxels, the intensity at X(x,y) is a function only of the speed (and hence gap width) of one leaf pair. But for a point moving periodically with period τ, amplitude *A*, initial phase $t_{0}$, the intensity received depends on both leaf and voxel motion. The intensity received at $X(t+t_{0};\tau ;A),\;\varphi_{p}$ is given by:

(1) $$\varphi_{p}=\int_{t=0}^{T}I(\chi_{rk}(t)-X(t+t_{0};\tau ;A))I(X(t+t_{0};\tau ;A)-\chi_{lk}(t))dt$$

where *T* is the total beam‐on‐time (in MU); I = 1 if its argument is positive (X(x,y) is exposed relative to that leaf) and is reduced by penumbra and leaf transmission for negative argument. For motion parallel to the leaves the leaf index, *k*, does not change with time. When motion is perpendicular, *k* varies with time and *y*. Target motion was assumed to be periodic. We used the single periodic function obtained from a typical clinical breathing trace (Fig. 1) for all simulations. Each respiratory cycle was divided into 13 equally‐spaced phases, and *A* and τ were scaled for investigation of the effects of amplitude and period on the dose. The target was assumed to be a rigid body, and changes in tissue inhomogeneity caused by tumor motion were not considered for the dose calculation.

### C. Calculations and analysis

The average ITV, CTV, and PTV were 9.9 (range, 1.5–27.3), 33 (range 10.2–75.7), and 70.7 (range 27.8–141.5) cc. Average field sizes were 7.7 (range 5–13) and 6.4 (range 4.5–8.5) cm parallel and perpendicular to leaf motion. The average ± standard deviation (σ) beam‐on‐time per beam was 1184±456 (range 513–2506) MU, and average ± σ leaf gap was 2.4±0.8 (range 0.7–3.5) cm. Depending on the breathing period, each treatment field included 13–36 breathing cycles.

Equation (1) was evaluated for dose prescriptions of 20 Gy × 1 and 20 Gy × 3 fractions using leaf‐sequence files from the original treatment plans, modified by the respiratory motion of Fig. 1 with respiratory amplitudes 0.36, 0.9 and 1.26 cm (peak‐to‐peak excursions 0.68, 1.7 and 2.38 cm), and periods of 3, 5, and 8 seconds. The initial respiration phase for each fraction was chosen randomly from points 1–13 in Fig. 1 and t in Eq. (1) was set to the corresponding MU. Doses calculated via Eq. (1) are denoted as Respiration Correlated Dose (RCD). To test the effects of initial respiratory phase, additional simulations were performing using three different starting phases: at the rising slope (Θ=1 in Fig. 1), maximum amplitude (Θ=5), and minimum amplitude (Θ=12). For a given treatment fraction, the same initial phase was used for each beam. $D_{\max}$, $D_{\text{min}}$, and $D_{\text{mean}}$ (maximum, minimum and average structure doses, respectively); $D_{95}$ and $D_{05}$ (dose encompassing the hottest 95% and 5% of the structure) and $V_{95}$ (percentage of structure receiving >95% of the prescribed dose) were calculated for the ITV and CTV. Ratios of all dosimetric parameters to the planned quantity, designated as $R_{\text{Dmax}}$, $R_{D95}$, etc, were also calculated. A ratio of 1.0 means that respiratory motion does not change the quantity; <1 means that it is reduced by motion, and >1 that it is increased. Although the PTV is a geometric construct that is not subject to motion, dose parameters were also calculated for the PTV to estimate the upper limit of respiration artifacts on delivered target dose.

## III. RESULTS

### A. Hypofractionated NSCLC plans

**Figure 2:**
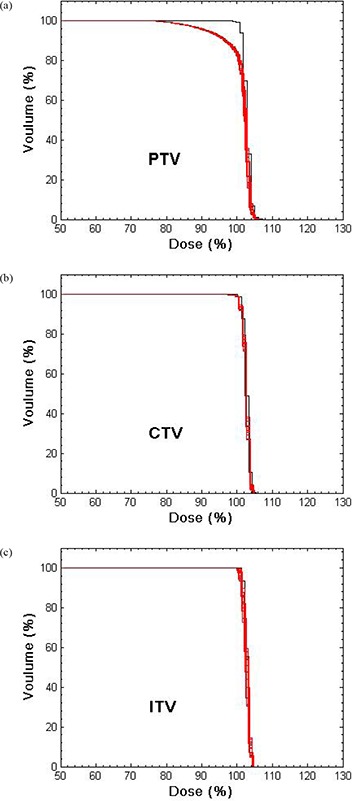
DVHs for the PTV, CTV and ITV for a lung IMHFRT treatment plan of 20 Gy × 1 fraction. Prescription dose corresponds to 100%.

The RCD was insensitive (<1%) to period and initial phase for all simulations, but amplitude effects could be large. Figure 2(a) compares the DVHs for the planned PTV, CTV and ITV (black curves) for a 20 Gy × 1 fraction treatment of Tumor #5 (4‐fields, average 1157 MU, 81.9, 40.2 and 13.0 cc for PTV, CTV and ITV) with the corresponding RCD (red curves) for A=0.9 cm, perpendicular to leaf motion. The red DVHs were generated by starting the motion at each of the 13 different phases shown in Fig. 1. The narrow spread of these DVHs shows the small statistical deviation due to different initial phases. The expectation value of the RCD lies within the bundle of red curves. For the PTV, it differs from the planned distribution, with dose uniformity degraded, and the sharp shoulder of the planned DVH rounded by respiration induced dose blurring at the field edges. Dose to the CTV is minimally reduced, while dose to the ITV is unchanged. Qualitatively similar motion effects were seen for parallel leaf and tumor motions.

**Figure 3:**
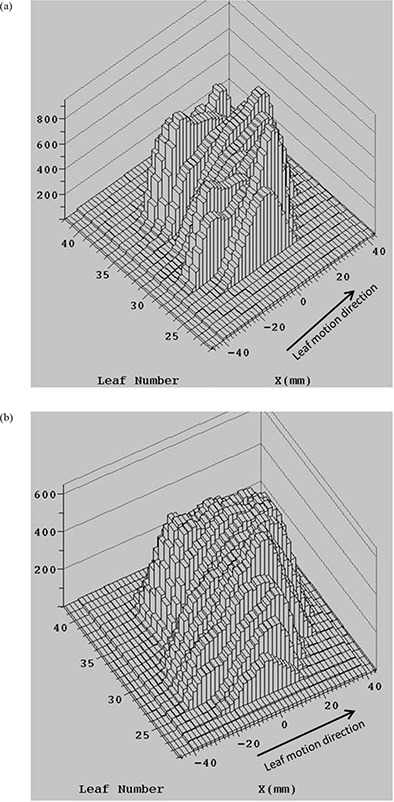
The planned intensity profile (a) and the effective intensity profile for respiratory motion with A=0.9 cm perpendicular to the leaf motion (b) for one lung HFRT field for single fraction.

Figure 3 shows the planned and the effective intensity profiles for one field from this case. Respiration‐induced smearing of the profile in the direction perpendicular to the leaf motion is evident.

**Figure 4:**
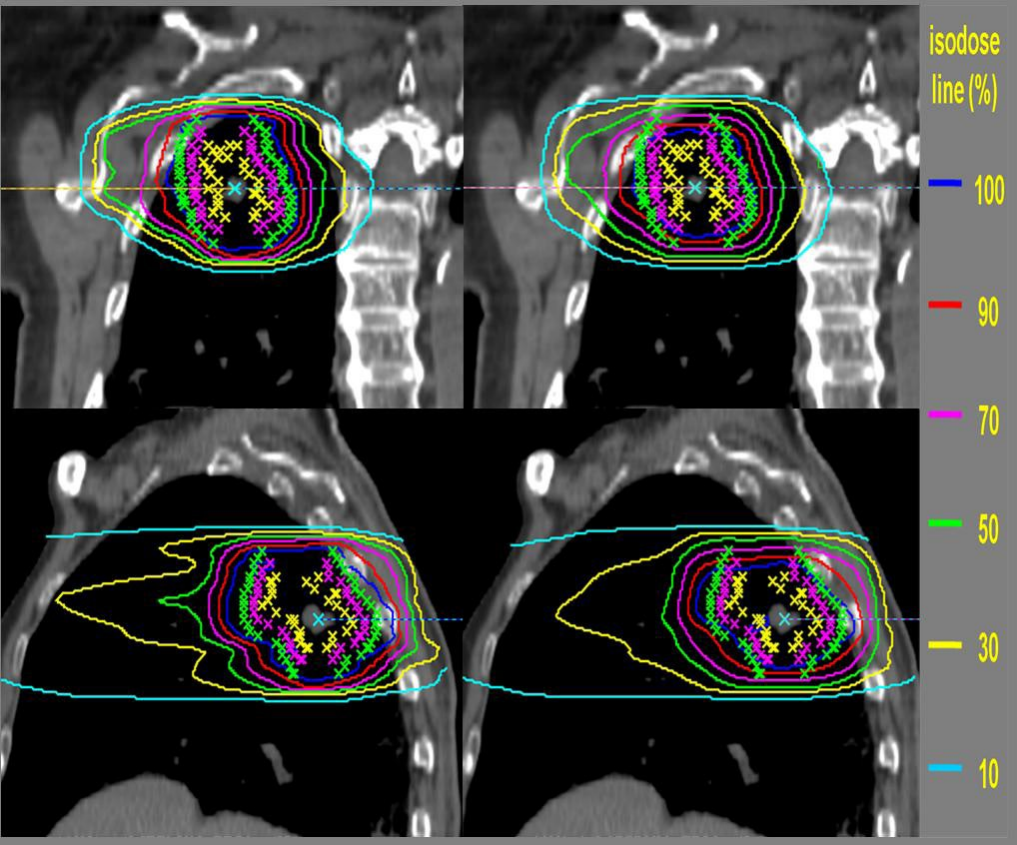
The isodose distributions for the static tumor (left panels) and moving tumor with A=0.9 cm perpendicular to leaf motion for 20 Gy × 3 fractions (right panels). Yellow, pink and green stars show the ITV, CTV and PTV, respectively. Lines are percentages of prescription isodose lines.

Figure 4 compares the planned and motion–affected isodose distributions for this case for three fractions (60 Gy) with randomly chosen initial phase at each fraction. Penumbra broadening in the cranial‐caudal direction and smoothing of the lower isodose lines are seen for the moving tumor. For organ motion parallel to leaf motion, the penumbra broadening is in the anterior‐posterior and left‐right directions. The penumbra broadening is responsible for the degradation of the PTV and CTV coverage.

**Figure 5:**
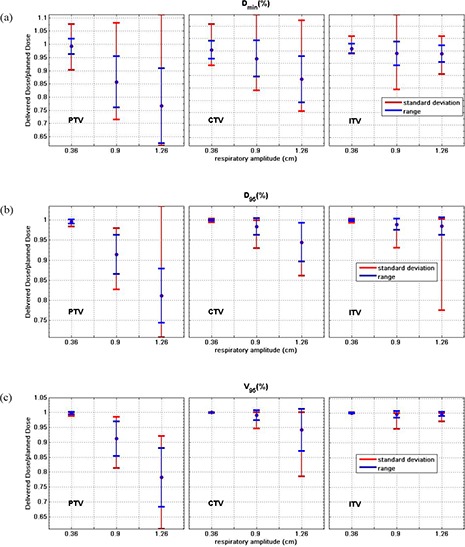
Average $R_{\text{Dmin}}$, $R_{D95}$ and $R_{V95}$ over 132 simulations of various motion periods and initial phases for moving tumor of all 11 HFRT treatment plans, PTV, CTV and ITV with the standard deviation (blue bars), and range (red bars) of the ratios. The y‐axis scaling does not include the complete range for the largest amplitude PTV results.

For each dosimetric parameter and amplitude, we averaged the RCDs calculated with the three different initial phases and three motion periods for 20 Gy × 1 and 20 Gy × 3 fractions over all tumors. Figure 5 shows averaged $R_{\text{Dmin}}$, $R_{D95}$ and $R_{V95}$ for A=0.36, 0.9, and 1.26 cm (perpendicular to leaf motion). Red error bars show the ranges of variation, blue error bars the standard deviations. $D_{\text{min}}$, $D_{95}$ and $V_{95}$ are sensitive to dose gradients at field edges or in regions of large modulation and are more sensitive to motion effects. Average $R_{\text{Dmin}}$ is 0.77 (range 0.62–1.11) and 0.89 (range 0.76–1.12) for the PTV and CTV for A=1.26 cm. Large motion amplitude could displace target edges beyond the aperture swept out by the leaves resulting, on average, in a reduction of $D_{\text{min}}$. Average $R_{\text{Dmin}}$ for the ITV is ≅ 0.98 (range 0.91–1.06), indicating little change in delivered dose. For the PTV, the average $R_{D95}$ and $R_{V95}$ with A=1.26 cm are reduced to 0.81 and 0.78. For the CTV, there is only a 6% reduction in $R_{D95}$ and $R_{V95}$, and for the ITV only 2% reduction, indicating that the CTV‐PTV margin chosen is sufficient.

Average ± standard deviation of $R_{D95}$ and $R_{V95}$ for A=1.26 cm for the PTV for six randomly selected patients are 0.8±0.06 and 0.75±0.10 for perpendicular, and 0.84±0.05 and 0.72±0.13 for parallel to leaf motions. These ratios show that the motion effects are qualitatively similar for perpendicular and parallel motions.

Changes in $D_{\text{mean}}$, $D_{\max}$ and $D_{05}$ are <2% for the ITV and CTV even for large amplitudes since most IMRT lung plans have relatively homogeneous dose distributions near the isocenter. Even for the PTV, decreases in $D_{\text{mean}}$ is <5%, and $D_{\max}$ and $D_{95}$ are <2%, similar to the observations of Chui et al.^(^ ^13^ ^)^ for conventional multi‐fractionated lung IMRT.

We also confirmed that for conventionally fractionated treatments these intensity distributions behaved similarly to those studied by others.^(^ ^12^ ^,^ ^13^ ^,^ ^15^ ^)^ Specifically, for a single 2 Gy fraction, interplay effects are more important and lead to greater dependence on initial phases, but the interplay effects average out for the 30 or more sessions that are typical for conventional fractionation.

### B. Highly‐modulated case

**Figure 6:**
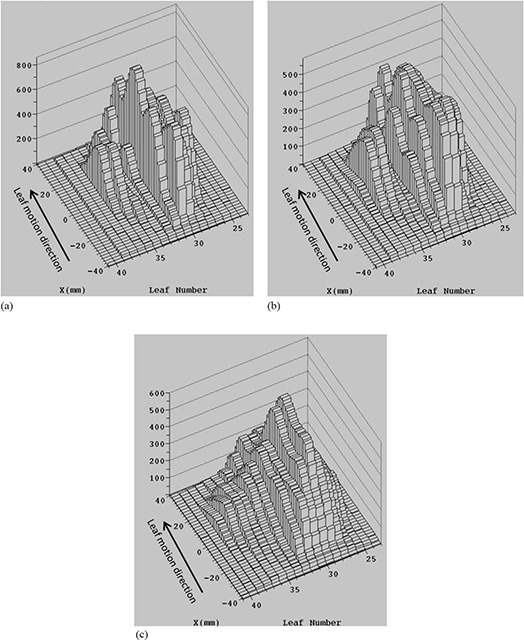
The intensity profile for static tumor (a) and effective intensity profile for a tumor moving with A=0.36 cm (b) and 0.9 cm (c) perpendicular to the leaf motion for the highly‐modulated intensity pattern for single fraction.

Finally we studied motion effects for a 20 Gy fraction of one highly‐modulated seven‐field treatment plan. The increased modulation was designed to protect a critical “serial” structure adjacent to the target volume. The planned and effective intensity profiles calculated from Eq. (1) for this intensity pattern differ greatly, as shown in Fig. 6. Blurring inside the field is increased because of larger differences in leaf motion profiles between adjacent leaf pairs.

**Figure 7:**
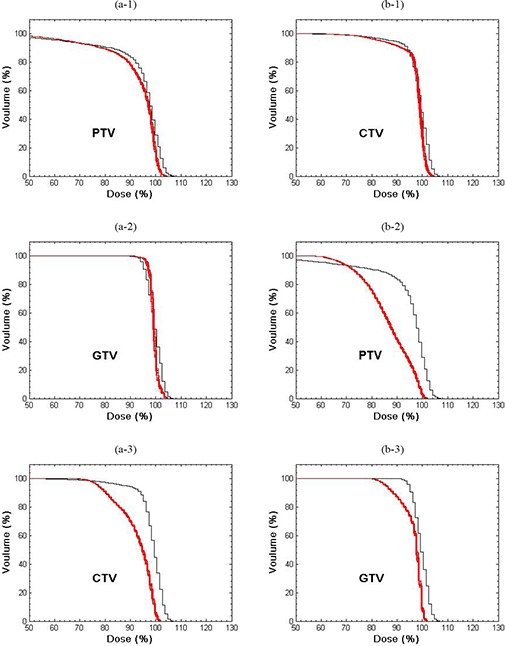
DVHs for the PTV, CTV and GTV for the highly‐modulated treatment plan for static tumor (black) and for 13 simulation of a tumor moving (red) with A=0.36 cm (a) and 0.9 cm (b). Prescription dose corresponds to 100%.

Figure 7 shows the DVHs for the planned dose and 13 simulations of the RCD with random initial phases for A=0.36 cm (Fig. 7(a)) and 0.9 cm (Fig. 7(b)), and τ=5sec. There is a noticeable change in the expectation value of the dose distribution to all structures (PTV, CTV, and GTV – taken to equal the ITV) even for small motion amplitude (Fig. 7(a)). However, there is little variation due to different initial phases (indicated by the small spread of the motion‐affected DVHs) because treatment extends over many breathing cycles per field, which averages out the interplay effects even for this highly‐modulated IMHFRT plan. The average $R_{\text{Dmean}}$ for A=1.28 cm are 0.89 and 0.92 for the CTV and GTV, with average $R_{\text{Dmax}}$ and $R_{D05}$ reduced to 0.94 and 0.95 for the CTV, and 0.95 and 0.96 for the GTV. The average $R_{D95}$ falls to 0.83 and 0.85 for the CTV and GTV. The average $R_{\text{Dmin}}$ and $R_{V95}$ are 0.80 and 0.41 for the GTV, and 1.38 and 0.19 for the CTV. These results indicate that expected values of dose depend strongly on the degree of modulation, but the interplay is small even for highly‐modulated intensity patterns with IMHFRT treatment.

## IV. DISCUSSION

When sliding window IMRT is delivered to a target that experiences respiratory motion, the effective beam intensity distribution is a complicated function of the tumor motion relative to that of the MLC leaves. For a single fraction at conventional dose and dose‐rate (2Gy, ~300–600 MU/min), several studies^(^ ^12^ ^,^ ^13^ ^,^ ^14^ ^,^ ^15^ ^,^ ^16^ ^)^ show that the dose distribution can depend on the initial breathing phase as well as the respiratory amplitude and period, but the variance of dose distribution from the many fractions is negligible. The study of Seco et al.^(^ ^16^ ^)^ implied that the overall dose error between delivered dose and the motion average dose for IMHFRT could be small due to long beam delivery time of high dose. Our study shows that for IMHFRT delivered with the sliding window technique at 600 MU/min or less, the variance has minimal dependence on respiratory period or initial phase. In our simulations, there are on the order of 10–50 breathing cycles per beam, thus mitigating any dependence on initial phases.

A key point is that the total beam‐on‐time for an entire course of treatment is approximately the same for IMHFRT (20 Gy × 3 fractions) and conventionally fractionated IMRT (2 Gy × 30 fractions). Thus the total number of breathing cycles during treatments is comparable which, in turn, results in similar variances for the RCD. This result is not, however, apparent a priori, but has been demonstrated by the simulations presented here. Further evaluation should be made for sliding window treatments delivered with higher dose rates (e.g. 1000 MU/min) and/or delivery methods that reduce the MU, especially in patients with naturally slow breathing periods or where 4DCT study shows large amplitude tumor motion. In such cases, the variance in daily delivered dose can be as large as it is for a single treatment with low dose (2 Gy/fraction) and, although motion affects average out over a full course of treatment, respiratory gating to limit the motion amplitude might be beneficial.

As discussed in previous studies, blurring effects depend on the proximity of the structure to the field edges and the degree of in‐field modulation. For modestly modulated NSCLC treatment plans and ~5 mm margins, these effects are most evident for the PTV while they are smallest for ITV, which is furthest from the field edge. $D_{\text{min}}$ for the CTV can be reduced by as much as 24% for large amplitude motion (e.g. 2.38 cm).

For more highly‐modulated fields than typically used for NSCLC treatments, respiratory motion blurring could be problematic. If future lung or other thoracic cases require highly‐modulated intensity patterns to protect a “serial” type normal structure (e.g. the esophagus or mainstem bronchus) respiration effects should be evaluated by the planner.

We have also not studied the effects of respiratory motion on other treatment techniques such as “step‐and‐shoot”, tomotherapy, or volumetric arc treatment. We also approximated tumor motion as one‐dimensional and periodic. In reality, it is three‐dimensional and often irregular. However, the weak dependence on period and phase, and the qualitative similarity of the effects of respiratory motion parallel and perpendicular to leaf motion, suggest that more accurate modeling for these factors would not change our conclusions. Finally, we did not account for deformation of the tumor or surrounding tissues.

It is well known that more advanced algorithms – superposition‐convolution or Monte Carlo – are preferable to the pencil beam algorithm for lung calculation.^(^ ^24^ ^)^ A more accurate study on tumor motion effects should be performed using these algorithms.

## V. CONCLUSIONS

Respiratory motion effects depend primarily on motion amplitude with negligible dependence on period or initial phase for the IMHFRT plans delivered for early‐stage NSCLC at our institution. For typical tumor geometries and respiratory amplitudes, changes in target coverage are minimal but can be significant for larger amplitudes, faster beam delivery, more highly‐modulated fields, and smaller field margins.

## ACKNOWLEDGEMENTS

This work was supported by a NIH training award T32‐CA61801.
